# *Bifidobacterium breve* alleviates dry eye disease by regulating gut microbiota and vitamin A metabolism

**DOI:** 10.3389/fnut.2026.1956357

**Published:** 2026-09-09

**Authors:** Liming Huang, Jinni Sun, Gao Tian, Chunwen Liu, Cong Li, Xueying Ma, Yurong Zhao, Zhangming Pei, Wenwei Lu, Hongchao Wang

**Affiliations:** 1 State Key Laboratory of Food Science and Resources, Jiangnan University, Wuxi, Jiangsu, China; 2 School of Food Science and Technology, Jiangnan University, Wuxi, Jiangsu, China; 3 Dong-E-E-Jiao Co., Ltd., Liaocheng, Shandong, China; 4 Dong'e Ejiao Health Industry Technology Co., Ltd., Liaocheng, Shandong, China; 5 National Engineering Research Center for Functional Food, Jiangnan University, Wuxi, Jiangsu, China

**Keywords:** *Bifidobacterium breve*, dry eye, gut flora, retinoic acid, vitamin A

## Abstract

The gut microbiota can metabolize vitamin A and may thereby alleviate symptoms of dry eye disease. *Bifidobacterium breve* CCFM1489 was investigated, a commensal strain capable of converting vitamin A (VA) into retinoic acid (RA), in a benzalkonium chloride-induced DED mouse model. The results demonstrated that CCFM1489 alleviated dry eye symptoms, as evidenced by reduced fluorescein sodium staining of the cornea, improved corneal and conjunctival histopathology. These effects were accompanied by reducing oxidative stress and inflammation whilst elevating systemic RA levels. Integration of gut metagenomic and faecal/serum metabolomic analyses provided preliminary insights into the underlying mechanisms. CCFM1489 reshaped the gut microbial composition and structure, increasing the abundance of specific taxa, including *Parabacteroides goldsteinii* and *Lactiplantibacillus plantarum*, that were associated with metabolic improvements. Functional shifts indicated enhanced microbial VA metabolism and modulation of host–microbiota pathways. Collectively, CCFM1489 regulates gut microbiota involved in VA metabolism and represents a promising microbiota-targeted strategy for ameliorating dry eye disease.

## 1 Introduction

Dry eye disease (DED) is a common multifactorial disorder of the ocular surface, affecting millions of individuals worldwide and imposing a substantial socioeconomic and quality-of-life burdens (1). Lifestyle shifts, particularly the widespread adoption of digital devices for both work and leisure, have driven a marked increase in DED incidence (2, 3). Sustained ocular surface dryness compromises the corneal epithelial barrier and depletes conjunctival goblet cells, clinically manifesting as itching, redness, and enhanced sensitivity to environmental irritants (4, 5). DED treatment strategies currently rely primarily on artificial tears, anti-inflammatory agents, or supplementation with vitamin A (VA) and retinoic acid (RA) (6–10). Despite their widespread use, these methods have significant limitations. Topical therapies typically have a short residence time on the ocular surface, requiring frequent administration, which may compromise patient compliance. Long-term usage of corticosteroids and anti-inflammatory medications might cause side effects include ocular surface toxicity or increased intraocular pressure (6, 11). Despite being vital for the integrity of the corneal and conjunctival epithelium, VA and RA have limited therapeutic efficacy due to their chemical instability, which makes them vulnerable to oxidative conditions, light, and heat (9). These challenges underscore the need for alternative strategies capable of modulating endogenous RA metabolism and systemic inflammatory status in a more sustained and physiologically relevant manner.

The intestinal microbiota comprises a diverse microbial community that participates in fundamental physiological functions, such as nutrient metabolism and immune homeostasis (12). With the emergence of the ‘gut–eye axis’ concept, increasing attention has been directed towards the role of gut microbial composition and microbial metabolites in ocular diseases (13, 14). Microbiota-derived metabolites can be absorbed in the intestine, enter systemic circulation, and reach distal tissues such as the ocular surface (15). Through these pathways, gut microbial activity can influence epithelial integrity, mucin production and inflammatory signaling at the ocular surface, thereby contributing to DED pathophysiology (16–19). An expanding body of research suggests that modulating gut microbiota to reshape microbial communities may help alleviate DED symptoms (4, 20). Previous studies have demonstrated that dietary supplementation with a prebiotic mixture comprising lactoferrin, fish oil, and *Enterococcus faecium* WB2000 confers significant relief from dry eye symptoms in both rodent models and clinical populations (21). In addition, IRT5, a probiotic consortium including *Bifidobacterium bifidum, Lactobacillus acidophilus, Lactobacillus casei, Lactobacillus reuteri*, and *Streptococcus thermophilus* ameliorated autoimmune DED and was associated with improvements in gut microbiota composition (22).

Accumulating evidence indicates that DED is associated not only with local ocular surface inflammation but also with systemic inflammatory responses and oxidative stress. Desiccating stress has been shown to induce T cell-mediated inflammatory responses, and inflammatory cytokines such as IL-17 play a critical role in disrupting corneal epithelial barrier function, reflecting both local and systemic inflammatory components in DED pathogenesis. These observations provide a rationale for evaluating serum inflammatory cytokines and corneal oxidative stress markers as indicators of systemic and local pathological changes in DED models. Therefore, in the present study, we assessed these parameters to evaluate the systemic and local effects of CCFM1489 intervention.

VA is an essential fat-soluble micronutrient that cannot be synthesised endogenously and must be obtained from the diet (23). RA, the principal bioactive form of VA, initiates downstream transcriptional programs through binding to RARs and RXRs, a process critical for corneal epithelial integrity and goblet cell homeostasis. Beyond its direct actions on the ocular surface, VA metabolism is profoundly influenced by the gut microbiota, which serves as a key endogenous regulator of retinoid bioavailability (24). Independent of the host, gut microorganisms can convert intestinal retinol and retinyl esters into bioactive metabolites such as retinal and RA (24). Similar to the host alcohol dehydrogenase/aldehyde dehydrogenase (ADH/ALDH) enzymatic pathway, gut bacteria possess analogous enzymatic activities that enable the conversion of VA into retinal or RA (25, 26). For instance, *Bacillus cereus* has been reported to contain ALDH enzymes functionally similar to human ALDH, capable of metabolising retinal into retinol and RA (25). Further studies have identified segmented filamentous bacteria, *B. bifidum*, *Lactobacillus intestinalis* and other intestinal microbes with similar ALDH activity that can produce RA from VA (24, 26, 27). Supplementation of these ALDH-active microbes into antibiotic-treated germ-free or germ-free mice restores intestinal RA levels and activates RAR-dependent signalling pathways in the host (24, 26, 27). These findings suggest that targeting VA-metabolising gut bacteria represents a promising strategy to enhance microbial RA production, promote host health and potentially relieve DED symptoms.

Among ALDH-containing bacteria, *Bifidobacterium breve* was selected in this study because its ALDH sequence shows the highest similarity to previously reported ALDHs from Bifidobacterium species (27). Moreover, *B. breve* is a well-established gut commensal with a long history of safe use in functional foods and clinical studies, making it a suitable and reliable candidate for probiotic intervention. Thus, to evaluate whether microbially derived RA can alleviate DED, we separately administered an ALDH-containing strain of *B. breve*, VA and its active metabolite RA to mice with benzalkonium chloride-induced dry eye. Oxidative stress, inflammatory responses and ocular surface integrity were assessed. Integrated gut metagenomic and faecal/serum untargeted metabolomic analyses were performed to explore the underlying mechanisms.

## 2 Materials and methods

### 2.1 Microbial strains and cultivation

All bacterial strains used in this study were sourced from the Culture Collection of Food Microorganisms (CCFM) at Jiangnan University (Wuxi, China). The strains were propagated through three successive passages in de Man–Rogosa–Sharpe (MRS) broth, after which the cells were harvested by centrifugation (8,000 rpm, 15 min, 4 °C), resuspended in 30% (v/v) glycerol, and stored at −80 °C until further use. *Bifidobacterium breve* CCFM1489 is preserved in the CCFM collection. Its purity was confirmed by Sanger sequencing, and its species identity was verified by whole-genome average nucleotide identity (ANI) analysis upon deposition. The genome sequence data have not been deposited in a public database but are available upon reasonable request by contacting the corresponding author or the CCFM center.

### 2.2 Probiotic metabolism of VA *in vitro*

To evaluate the ability of selected strains to metabolise VA, an in *vitro* assay was conducted following previously described methods with minor modifications (24, 27). Activated strains were incubated anaerobically at 37 °C for 13 h. A VA/dimethyl sulphoxide (DMSO) solution (prepared by dissolving 100 mg VA in 4 mL DMSO) was then added to the cultures to reach a final concentration of 100 μmol/L. After 3 h of incubation, cultures were centrifuged and the supernatants were transferred into amber tubes and stored at −80 °C for subsequent analysis.

### 2.3 Experimental animals

Six-week-old male C57BL/6 J mice were obtained from Vital River Laboratory Animal Technology Co., Ltd. (Jiaxing, China) and maintained under specific pathogen-free (SPF) conditions. The environment was controlled at 20–26 °C with 40–70% relative humidity and a 12 h light/dark cycle. All mice had ad libitum access to food and water. All mice had ad libitum access to food and water. The mice were fed a standard commercial chow diet (Co60-irradiated growth and breeding diet for experimental mice) manufactured by Jiangsu Xietong Pharmaceutical Bio-engineering Co., Ltd. (Nanjing, China), which contains approximately 14,000 IU/kg of VA according to the manufacturer’s specifications. The study was approved by the Animal Ethics Committee of Jiangnan University (JN.No20241215c1500220[642]).

After a 1-week acclimation period, 40 mice were randomly assigned to five groups (*n* = 8 per group): control, model, VA, RA and CCFM1489 intervention groups. DED was induced according to previously described methods (28, 29). Briefly, the 21-day experiment included a 7-day acclimation period, 7-day modelling period and 7-day intervention period. During modelling, control mice received 5 μL saline in both eyes twice daily, whilst all other groups received 5 μL of 0.2% BAC twice daily.

During the intervention period, all mice received oral gavage in the morning and afternoon. The control and model groups received 200 μL saline and 200 μL vegetable oil daily. The VA group received 200 μL saline plus 200 μL experimental-grade corn oil (Meilunbio, China, MB1148-1) containing 0.5 mg VA (30, 31). The RA group received 200 μL saline plus 200 μL experimental-grade corn oil containing 0.5 mg RA (30, 31). The CCFM1489 group received 200 μL of bacterial suspension (live *B. breve* CCFM1489 resuspended in sterile saline to 1 × 109 CFU/mL) along with 200 μL experimental-grade corn oil. The Control and Model groups received equal volumes of saline and the same batch of experimental-grade corn oil by gavage.

### 2.4 Corneal fluorescein sodium staining

On day 7 of the intervention, 5 μL of fluorescein sodium solution (0.125% w/v) was applied to the conjunctival sac. Corneal integrity was assessed and photographed using a portable slit lamp equipped with a cobalt blue filter (32). For scoring, the cornea was divided into four quadrants. Each quadrant was scored on a 0–4 scale: 0, no staining; 1, 1–30 punctate staining points; 2, more than 30 punctate staining points without coalescence; 3, diffuse or filamentous staining; and 4, positive fluorescein plaque staining. The scores of the four quadrants were summed to obtain the final score for each mouse.

### 2.5 Histopathological analysis of corneal and conjunctival tissues

Eyeballs were fixed in 4% paraformaldehyde, embedded in paraffin, and sectioned. Corneal morphology was examined by haematoxylin and eosin (H&E) staining, whilst conjunctival goblet cells were evaluated using periodic acid–Schiff (PAS) staining. The Image J software was used to calculate the number of goblet cells in the conjunctiva in histological sections.

### 2.6 Enzyme-linked immunosorbent assay

Commercially available ELISA kits (Shanghai Enzyme-linked Biotechnology Co., Ltd., Shanghai, China) were utilized for the quantitative determination of MDA, SOD, and GSH-Px in corneal tissues, and of IL-6, TNF-α, IL-17, and RA in serum samples. All procedures were carried out strictly following the manufacturer’s recommended protocols.

### 2.7 Untargeted metabolomics analyses

Untargeted metabolomics of serum and faecal samples was performed by Novogene Co., Ltd. (Beijing, China) (33, 34). The mixtures were incubated on ice for 5 min and centrifuged at 15,000 × g for 20 min at 4 °C. The supernatants were then diluted with LC–MS-grade water to a methanol concentration of 53% (v/v), re-centrifuged, and finally transferred into glass autosampler vials for UHPLC–MS/MS analysis.

For untargeted metabolomic profiling, UHPLC–MS/MS analysis was conducted on a Vanquish UHPLC system interfaced with an Orbitrap Q Exactive™ HF, HF-X, or Exploris™ 120 mass spectrometer (Thermo Fisher Scientific, Dreieich, Germany). Samples were separated on a Hypersil Gold column (100 × 2.1 mm, 1.9 μm particle size) at a flow rate of 0.2 mL/min, using a linear gradient over 12 min. The mobile phase system consisted of 0.1% (v/v) formic acid in water (eluent A) and methanol (eluent B). The gradient was programmed as follows: 2% B at 0–1.5 min; a linear increase from 2 to 85% B at 1.5–4.5 min; a further increase from 85 to 100% B at 4.5–10 min; a decrease to 2% B at 10–10.1 min; and re-equilibration at 2% B from 10.1 to 12 min. The mass spectrometer was operated in both positive and negative electrospray ionisation modes, with the following instrumental settings: spray voltage, 3.5 kV; capillary temperature, 320 °C; sheath gas pressure, 35 psi; auxiliary gas flow, 10 L/min; S-lens RF level, 60; and auxiliary gas heater temperature, 350 °C.

The sample preparation protocol did not include a saponification step; therefore, the reported retinoid concentrations represent the levels of free (unesterified) RA and its metabolites. It should be noted that a portion of the detected retinoids corresponds to retinol (VA), reflecting the *in vivo* metabolic interconversion between retinol and retinoic acid.

### 2.8 Metagenomic analysis of mousefeces

Metagenomic sequencing was performed on the Illumina NovaSeq 6000 platform (Novogene, Beijing, China). Raw reads were processed using Trimmomatic (version 0.36) to remove low-quality sequences, retaining only those longer than 60 bp with an average quality score ≥30 (35). Clean reads were subsequently mapped to the mouse reference genome (GRCm38) using Burrows-Wheeler Aligner (BWA), SAMtools, and BEDTools to remove host-derived sequences (35).

### 2.9 Statistical analysis

GraphPad Prism (version 9.5.0; GraphPad Software, San Diego, CA, USA) and the R statistical computing environment (version 4.3.1; R Foundation for Statistical Computing, Vienna, Austria) were employed for data processing, statistical analysis, and graphical visualisation. Multivariate discriminant analyses, including OPLS-DA and PLS-DA, were performed using the ropls package (version 1.34.0) in R. All quantitative variables are expressed as mean ± standard error of the mean (SEM). For multiple-group comparisons, one-way analysis of variance (ANOVA) was applied, followed by Dunnett’s test for *post-hoc* comparisons against the MOD group, which inherently includes built-in correction for multiple comparisons. Statistical significance relative to the model group is denoted as follows: *p < 0.05, **p < 0.01, ***p < 0.001, and ****p < 0.0001; other comparisons are specified in the figure legends or text where applicable.

## 3 Results

### 3.1 *Bifidobacterium breve* CCFM1489 efficiently metabolises VA to produce high levels of retinoic acid

Previous studies have identified ALDH homologues in *B. cereus* that resemble human ALDH genes, leading to the discovery of gut microorganisms capable of converting VA into RA. Supplementation with gut microbes harbouring such ALDH genes has been proposed to enhance microbial VA metabolism and confer health benefits (24, 27, 36).

In this study, BLASTP analyses were performed comparing the protein structures of *B. cereus* ALDH1A1 (KFL74159.1) and *B. bifidum* ALDH (WP_015438559.1) with draft genomes of strains preserved in the culture collection at the School of Food Science and Technology, Jiangnan University, to identify ALDH-containing candidates. A group of *B. breve* strains with high amino acid sequence similarity was selected for further evaluation, and the comparison results are summarised in Supplementary Table S1.

**Figure 1:**
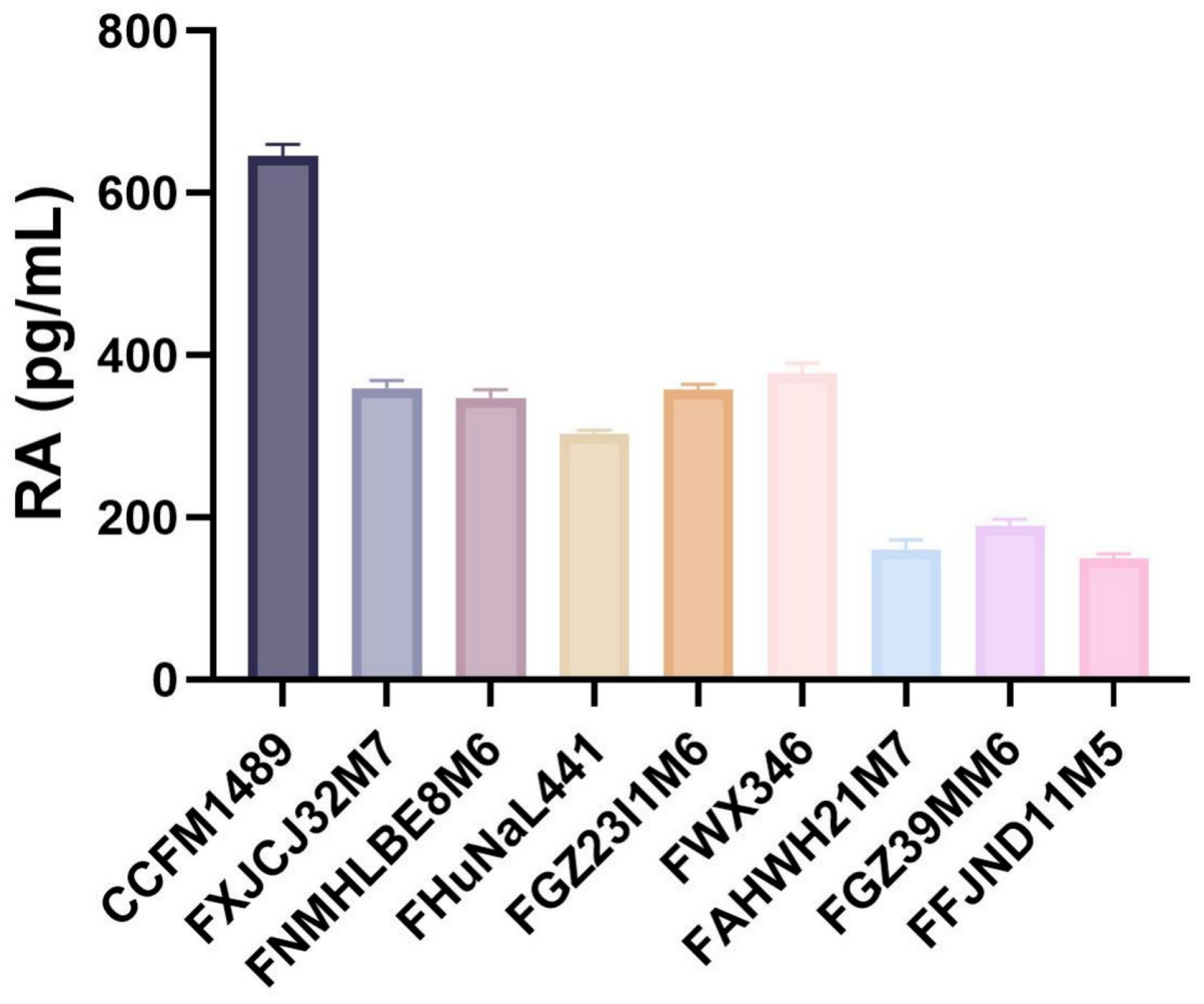
*Bifidobacterium breve* CCFM1489 efficiently converts vitamin A into retinoic acid. Data are presented as mean ± SEM (*n* = 3 independent experiments). Statistical significance was determined using one-way ANOVA followed by Dunnett’s test.

Consistent with previous findings, we confirmed that gut microorganisms carrying ALDH genes can metabolise VA into RA. Among the screened strains, *B. breve* CCFM1489 produced the highest concentration of RA when supplied with VA (Figure 1).

### 3.2 *Bifidobacterium breve* CCFM1489 alleviates DED

**Figure 2:**
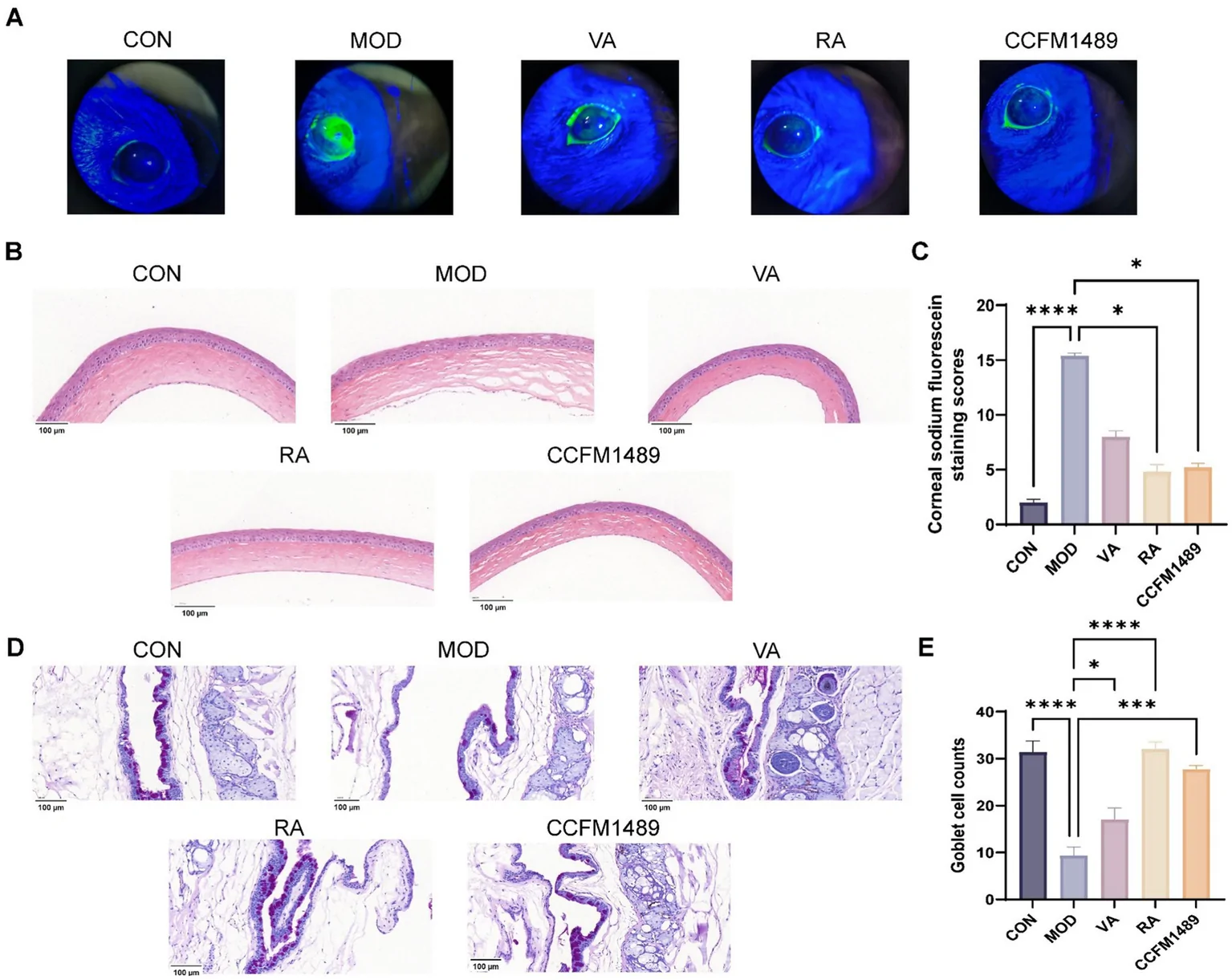
*Bifidobacterium breve* CCFM1489 alleviates dry eye disease in mice. **(A)** Representative slit-lamp images of sodium fluorescein-stained corneas. **(B)** Representative haematoxylin and eosin (H&E)-stained sections of the cornea (magnification, ×40). **(C)** Corneal sodium fluorescein staining scoring. **(D)** Representative periodic acid–Schiff (PAS)-stained sections of the conjunctiva (magnification, ×30). **(E)** Quantitative analysis of the counts of goblet cells in the conjunctiva. Data are presented as mean ± SEM (*n* = 8 mice per group for all panels). Statistical significance was determined using one-way ANOVA followed by Dunnett’s test for comparisons against the MOD group. *p < 0.05, **p < 0.01, ***p < 0.001, and ****p < 0.0001; ns, not significant.

To assess corneal epithelial damage, sodium fluorescein staining was performed on the corneas of dry eye model mice (37). Corneal epithelial barrier compromise, together with conjunctival goblet cell loss, represents a well-documented histological feature of DED. As shown in Figure 2, compared with the untreated control group, mice that received benzalkonium chloride twice daily for 1 week developed pronounced punctate corneal staining, along with basal epithelial vacuolisation, and a marked decrease in conjunctival goblet cells in tissue sections. These findings indicate severe pathological injury to both the corneal and conjunctival tissues.

Among all intervention strategies, treatment with either RA or *B. breve* CCFM1489 markedly alleviated DED symptoms. The extent of corneal staining was substantially reduced, and improvements were observed in corneal thickness, epithelial vacuolisation, and goblet cell numbers, demonstrating significant restoration of ocular surface integrity.

### 3.3 *Bifidobacterium breve* CCFM1489 increases retinoic acid levels in mice

**Figure 3:**
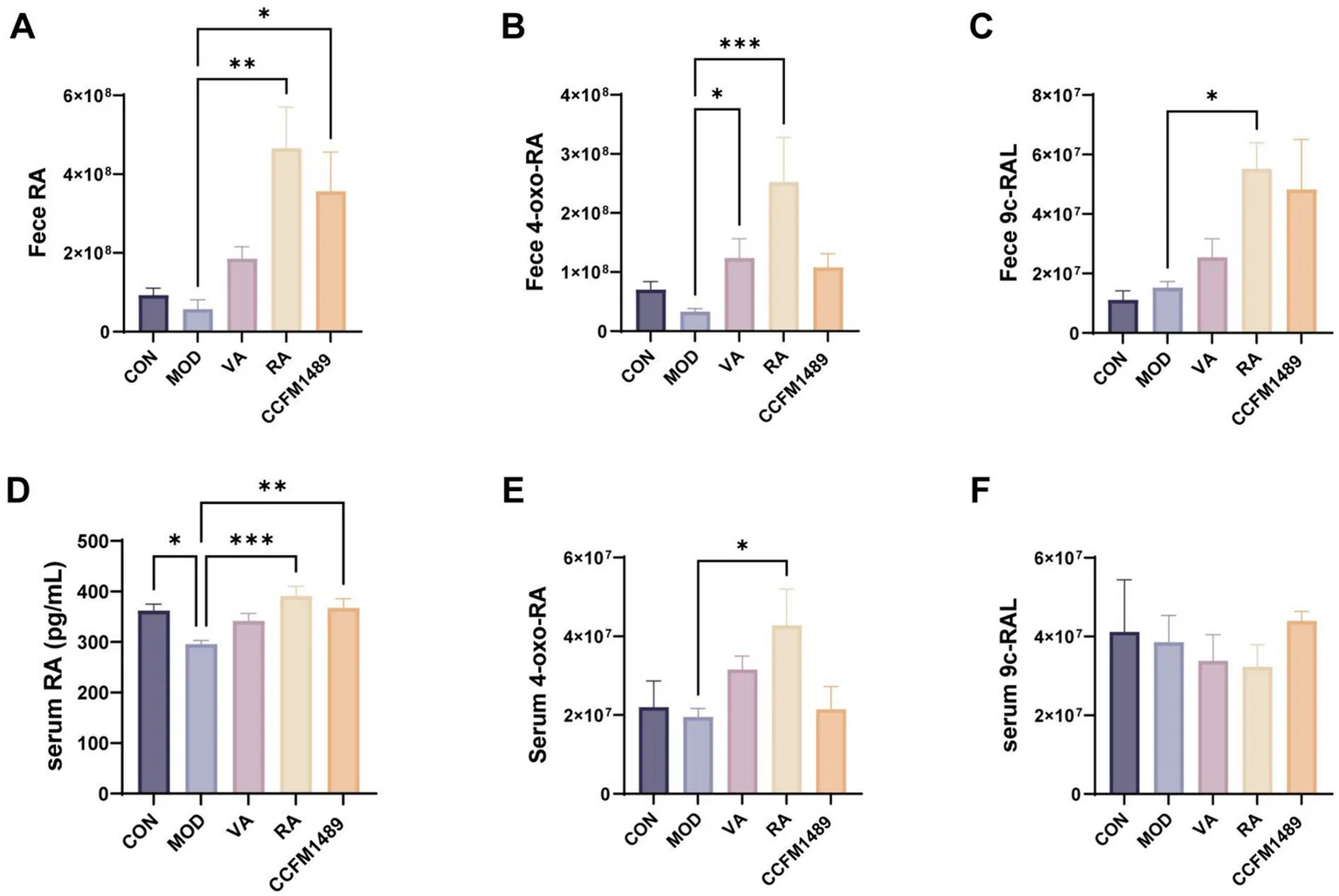
*Bifidobacterium breve* CCFM1489 enhances retinoic acid concentration in mice. **(A)** Faecal retinoic acid levels. **(B)** Faecal 4-oxo-retinoic acid levels. **(C)** Faecal 9-cis-retinal levels. **(D)** Serum retinoic acid levels. **(E)** Serum 4-oxo-retinoic acid levels. **(F)** Serum 9-cis-retinal levels. Statistical significance was determined using one-way ANOVA followed by Dunnett’s test for comparisons against the MOD group. *p < 0.05, **p < 0.01, ***p < 0.001, and ****p < 0.0001; ns, not significant.

The levels of retinoid metabolites were evaluated in mouse feces and serum. Oral administration of VA increased the levels of most retinoid metabolites except 4-oxo-RA, although these changes were not statistically significant (Figures 3A–F). In contrast, oral RA supplementation significantly elevated faecal levels of RA, 4-oxo-RA, and 9-cis-retinal, as well as serum levels of RA and 4-oxo-RA, compared with the model group. Following intervention with *B. breve* CCFM1489, RA levels in bothfeces and serum of dry eye mice were markedly increased, indicating effective enhancement of systemic RA status.

### 3.4 *Bifidobacterium breve* CCFM1489 reduces inflammatory cytokines and oxidative stress markers in mice

**Figure 4:**
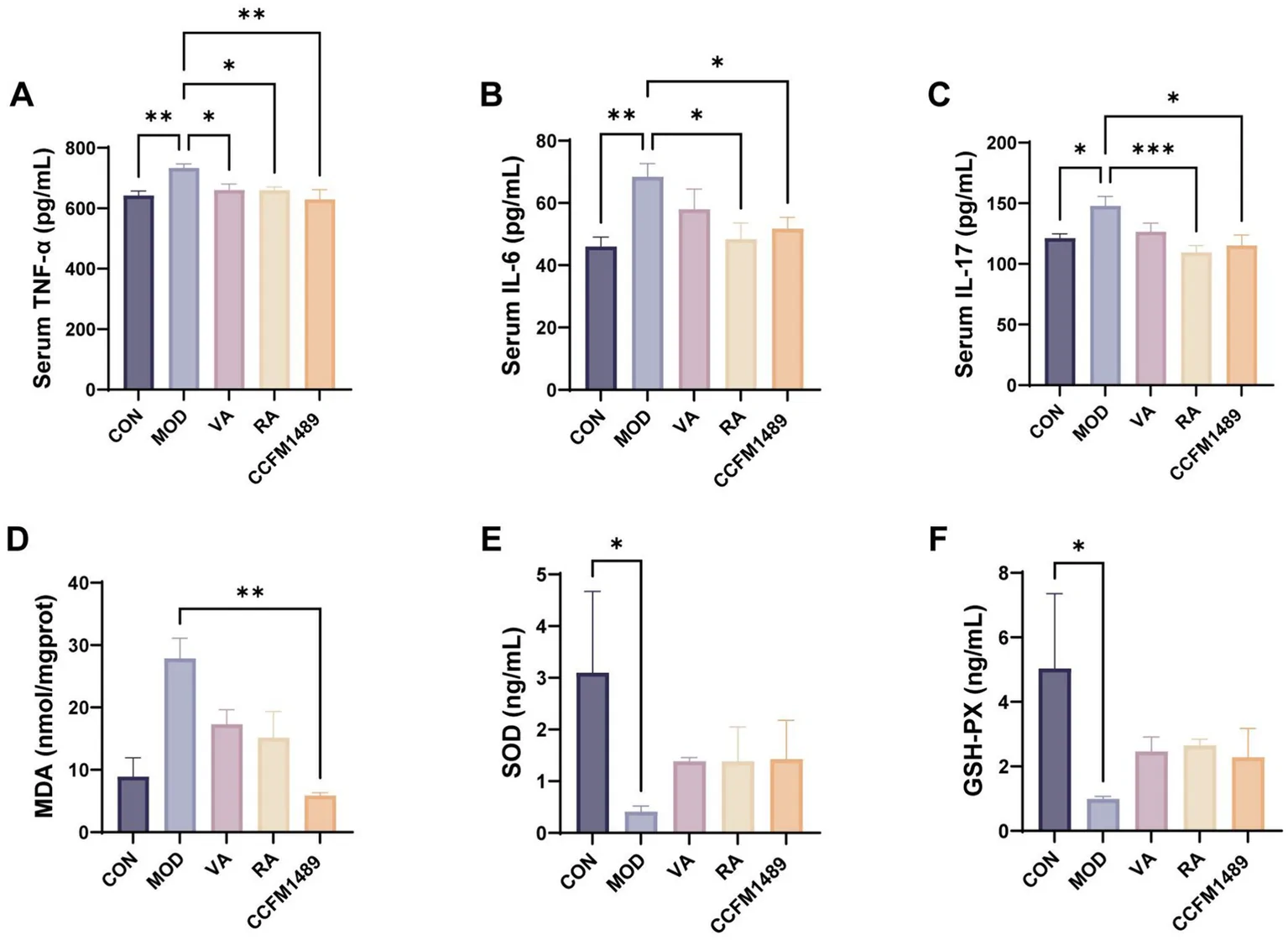
*Bifidobacterium breve* CCFM1489 improves physiological indicators in mice. Serum concentrations of **(A)** TNF-α, **(B)** IL-6, and **(C)** IL-17 in mice. Corneal levels of **(D)** MDA, **(E)** SOD, and **(F)** GSH-Px in mice. Statistical significance was determined using one-way ANOVA followed by Dunnett’s test for comparisons against the MOD group. *p < 0.05, **p < 0.01, ***p < 0.001, and ****p < 0.0001; ns, not significant.

We assessed serum cytokine levels and corneal oxidative stress markers in mice. Compared with the control group, dry eye mice exhibited significantly elevated serum concentrations of TNF-α, IL-6, and IL-17 (Figures 4A–C), alongside increased corneal MDA levels and decreased activities of antioxidant enzymes, including superoxide dismutase SOD and GSH-Px (Figures 4D–F).

Oral administration of RA or *B. breve* CCFM1489 significantly reduced serum TNF-α, IL-6, and IL-17 concentrations in dry eye mice (Figures 4A–C). Furthermore, CCFM1489 intervention markedly decreased corneal MDA levels (Figure 4D), indicating restoration of redox homeostasis and attenuation of inflammatory responses.

### 3.5 Effects of *Bifidobacterium breve* CCFM1489 on the gut microbiota of mice

**Figure 5:**
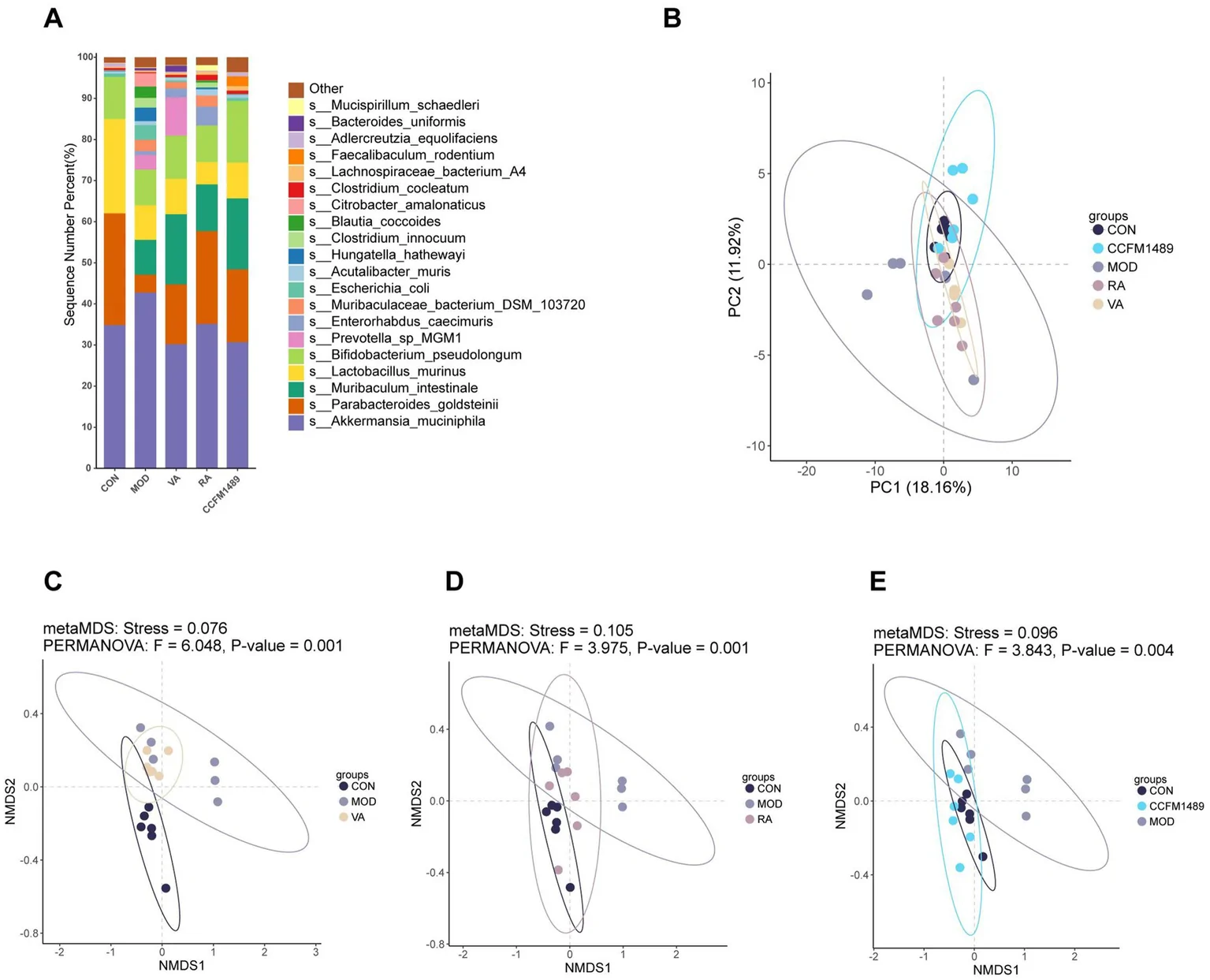
*Bifidobacterium breve* CCFM1489 modulates mouse gut microbiota. **(A)** Species-level relative abundance of gut microbiota. **(B)** PCA of gut microbiota at the species level. **(C–E)** Bray–Curtis NMDS analysis of gut microbiota. Data are presented as mean ± SEM (*n* = 8 mice per group). Statistical significance for NMDS was assessed using PERMANOVA with 999 permutations. *p < 0.05 was considered statistically significant.

Metagenomic analysis was performed to characterise faecal gut microbiota composition in mice. A cumulative relative abundance bar plot of the top 20 species is presented in Figure 5A. At the species level, compared with the model group, the abundance of *Alistipes indistinctus* tended to increase in the other intervention groups. Additionally, mice receiving RA or *B. breve* CCFM1489 exhibited higher abundances of *Clostridium cochleatum* compared with other groups.

*β*-Diversity of the gut microbiota was assessed using non-metric multidimensional scaling (NMDS) based on Bray–Curtis distances and principal component analysis (PCA). PCA revealed that, except for the model group, the microbiota structures of the other intervention groups were similar, with the CCFM1489 group most closely resembling that of the control group (Figure 5B). NMDS analysis further indicated significant differences between the model group and the VA, RA, and CCFM1489 groups (Figure 5C).

**Figure 6:**
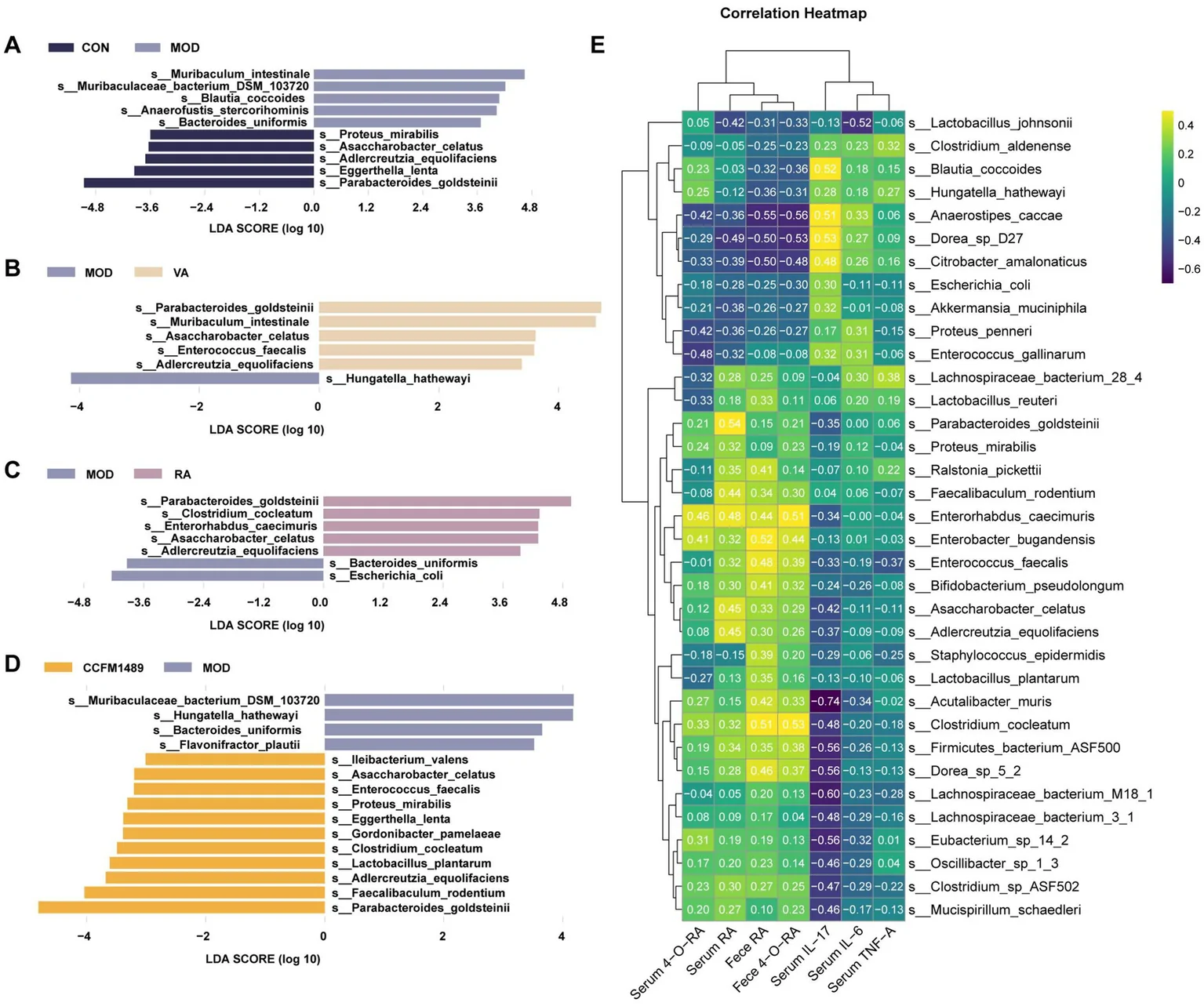
*Bifidobacterium breve* CCFM1489 modulates mouse gut microbiota. Results of LEfSe (LDA > 3.0) for **(A)** CON, **(B)** VA, **(C)** RA, and **(D)** CCFM1489 groups compared with MOD group, showing the differentially abundant species enriched in each group. **(E)** Spearman correlation analysis between the relative abundance of the top 35 gut microbial species and physiological parameters. The numerical values in the heatmap represent the Spearman’s rank correlation coefficient (*ρ*), with positive values indicating a positive correlation and negative values indicating a negative correlation. Data are presented as mean ± SEM (*n* = 8 mice per group). Statistical significance for LEfSe was determined using the Kruskal–Wallis test followed by pairwise Wilcoxon tests (LDA score > 3.0). For Spearman correlation analysis, *p < 0.05 was considered statistically significant.

Linear discriminant analysis effect size (LEfSe) analysis was conducted to identify key differential microbial taxa amongst groups (linear discriminant analysis [LDA] > 3.0). In the control group, the abundances of *Proteus mirabilis, Asaccharobacter celatus, Adlercreutzia equolifaciens*, *Parabacteroides goldsteinii*, and *Eggerthella lenta* were increased (Figure 6A). In the VA group, *P. goldsteinii*, *Muribaculum intestinale, A. celatus*, *E. faecalis*, and *A. equolifaciens* were enriched (Figure 6B). The RA group exhibited increased abundances of *A. equolifaciens, A. celatus, Enterorhabdus caecimuris*, *Clostridium cocleatum*, and *P. goldsteinii* (Figure 6C). In the CCFM1489 group, abundances of *Ileibacterium valens, A. celatus, E. faecalis, P. mirabilis, E. lenta, Gordonibacter pamelaeae, P. goldsteinii, C. cocleatum, Lactiplantibacillus plantarum, Faecalibaculum rodentium*, and *A. equolifaciens* were increased (Figure 6D). Notably, both the RA and CCFM1489 groups exhibited upregulation of beneficial species *P. goldsteinii* and *C. cocleatum* (Figures 6C,D), whilst the CCFM1489 group additionally increased the abundance of *L. plantarum*.

Spearman correlation analysis between microbial abundances and physiological parameters revealed that faecal RA was positively correlated with *Bifidobacterium pseudolongum, C. cocleatum*, and *E. faecalis*, whereas serum RA was positively correlated with *A. equolifaciens, A. celatus, F. rodentium*, and *P. goldsteinii* (Figure 6E). Moreover, *L. plantarum* showed a positive correlation with faecal RA levels.

**Figure 7:**
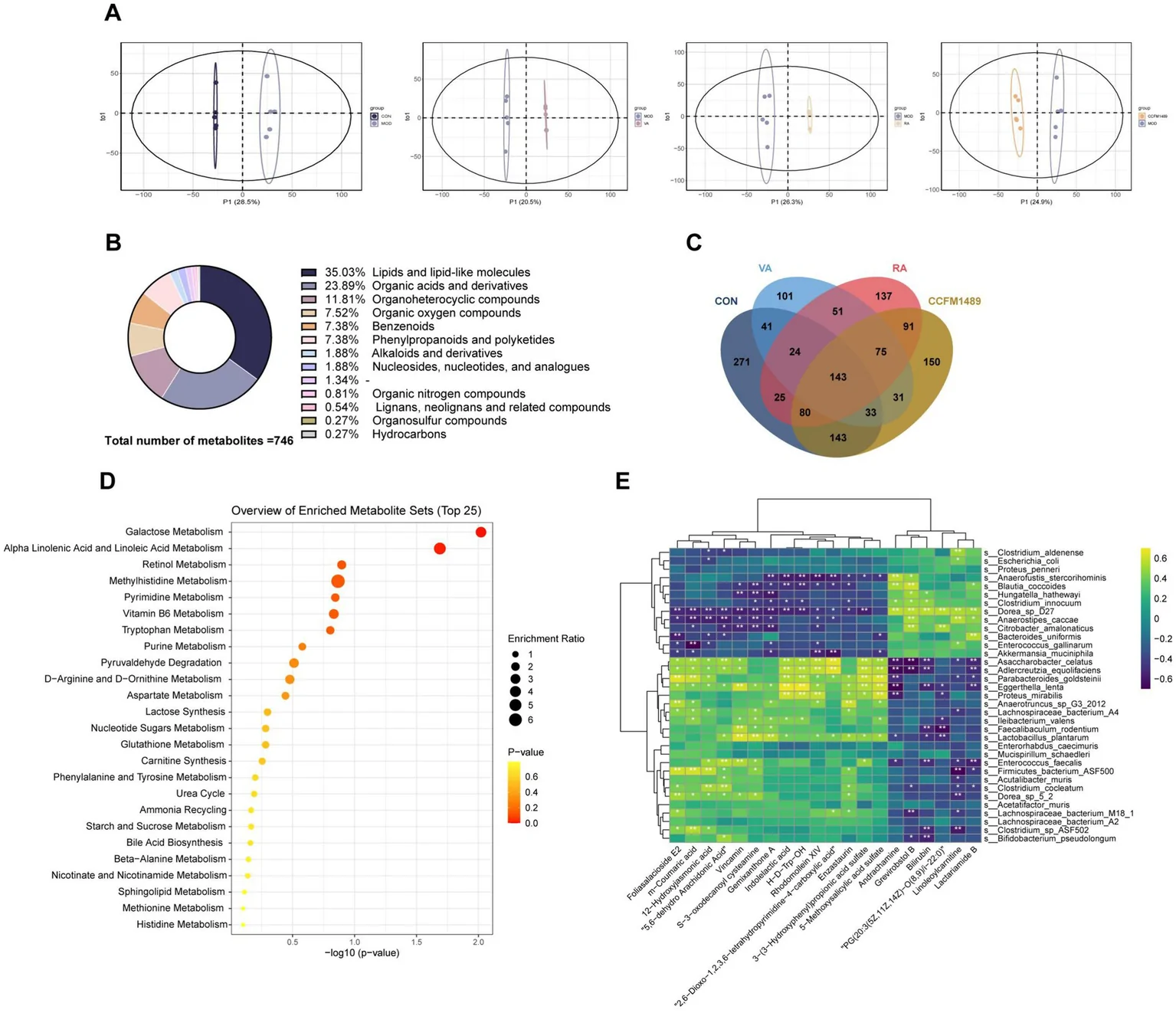
*Bifidobacterium breve* CCFM1489 modulates mouse faecal metabolome. **(A)** OPLS-DA results of all groups compared with MOD. **(B)** Differential metabolites between CCFM1489 and MOD groups classified into superclasses. **(C)** Common metabolites amongst metabolic sets revealed by Venn diagram. **(D)** KEGG functional pathway analysis of enriched differential metabolites in CCFM1489 and MOD groups, showing the top 25 pathways. **(E)** Spearman correlation analysis of the top 20 common differential metabolites with the top 35 gut microbes. Data are presented as mean ± SEM (*n* = 6 mice per group for metabolomics analyses). Statistical significance for OPLS-DA was assessed using permutation tests (200 permutations). Spearman correlation analysis was performed with *p < 0.05 considered statistically significant.

Restructuring of the gut microbiota can lead to alterations in intestinal metabolites. To investigate changes in the faecal metabolome of mice, untargeted metabolomics analysis was performed. OPLS-DA was applied to compare the metabolite profiles of all intervention groups with those of the model group. The OPLS-DA results indicated significant differences in metabolite phenotypes between each intervention group and the model group (Figure 7A). To ensure model validity, permutation tests were conducted, confirming the absence of overfitting and demonstrating the predictive reliability of the model (Supplementary Figure S2).

Differential metabolites between groups were identified based on a variable importance in projection (VIP) score >1 and |fold change| (FC) > 2. Differential metabolites in the CCFM1489 group compared with the model group were classified according to Human Metabolome Database (HMDB) SuperClass categories (Figure 7B). To explore altered metabolic pathways and their biological significance, enriched differential metabolites between the CCFM1489 group and the model group were subjected to KEGG pathway enrichment analysis, with the top 25 pathways shown (Figure 7D). These differential metabolites were predominantly enriched in galactose metabolism, linoleic acid metabolism, retinol metabolism, pyrimidine metabolism, and vitamin B6 metabolism (Figure 7D). Additionally, enrichment was observed in tryptophan metabolism.

Spearman correlation analysis between gut microbial abundances and common differential metabolites (*n* = 143) revealed significant positive correlations between *A. equolifaciens*, *A. celatus, P. goldsteinii, L. plantarum*, and indolelactic acid (Figure 7E).

### 3.6 *Bifidobacterium breve* CCFM1489 modulates the serum metabolome in mice

**Figure 8:**
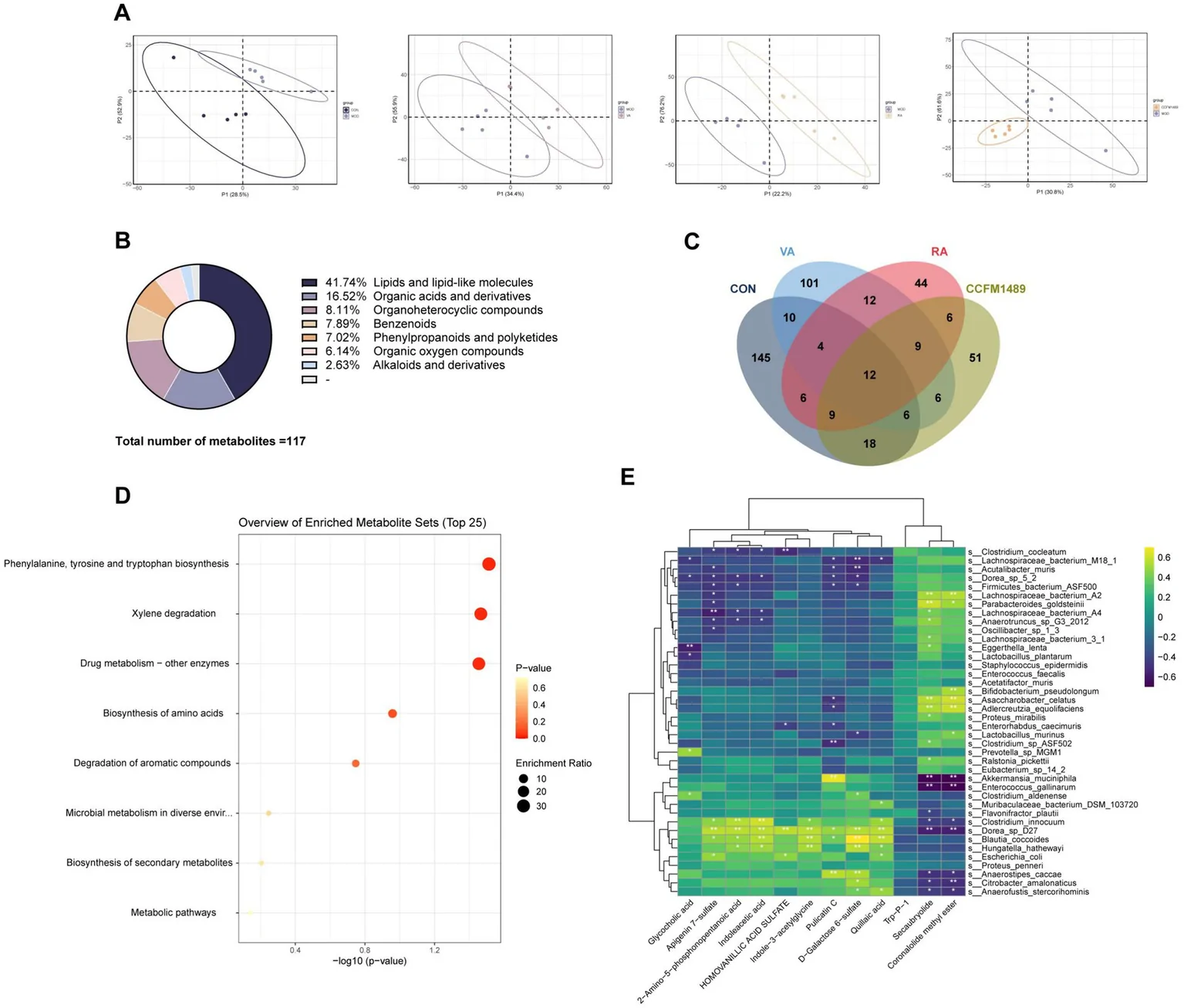
*Bifidobacterium breve* CCFM1489 modulates the mouse serum metabolome. **(A)** PLS-DA analysis results of all groups compared with MOD. **(B)** Differential metabolites between CCFM1489 and MOD groups classified into superclasses. **(C)** Common metabolites amongst metabolic sets revealed by Venn diagram. **(D)** KEGG functional pathway analysis of enriched differential metabolites in CCFM1489 and MOD groups, showing the top 8 enriched pathways. **(E)** Spearman correlation analysis of the top 12 common differential metabolites with the top 40 gut microbes. The numerical values in the heatmap represent the Spearman’s rank correlation coefficient (*ρ*), with positive values indicating a positive correlation and negative values indicating a negative correlation. Data are presented as mean ± SEM (*n* = 6 mice per group for metabolomics analyses). Statistical significance for PLS-DA was assessed using permutation tests (200 permutations). Spearman correlation analysis was performed with *p < 0.05 considered statistically significant.

To further investigate how *B. breve* CCFM1489, VA, and RA influence systemic metabolism, untargeted metabolomics analysis was performed on mouse serum. Partial least squares discriminant analysis (PLS-DA) was applied to assess metabolite profiles across all groups. The results showed that the serum metabolite profiles of the RA and CCFM1489 groups differed significantly from those of the model group (Figure 8A; Supplementary Figure S3).

Differential metabolites were selected based on variable importance in projection (VIP) scores greater than 1 and absolute fold change (|FC|) values exceeding 2. A total of 117 serum metabolites showing differential abundance between the CCFM1489 and model groups were categorized according to the Human Metabolome Database (HMDB) SuperClass classification system (Figure 8B). Further KEGG pathway enrichment analysis of serum differential metabolites revealed predominant enrichment in biosynthesis of amino acids, degradation of aromatic compounds, phenylalanine, tyrosine and tryptophan biosynthesis (Figure 8D; Supplementary Table S5).

Spearman correlation analysis between gut microbial abundances and common differential metabolites (*n* = 12) indicated that indoleacetic acid and indole-3-acetylglycine were significantly negatively correlated with *C. cocleatum*, whilst *L. plantarum* exhibited significant negative correlation with glycocholic acid (Figure 8E).

## 4 Discussion

In this study, a BAC-induced DED model was established in C57BL/6 J mice using topical administration of 0.2% BAC for seven consecutive days. BAC is a widely used ophthalmic preservative with well-documented cytotoxic, pro-apoptotic and pro-inflammatory properties, which disrupt tear film stability and compromise corneal epithelial integrity (38, 39). Consistent with previous reports, the selected concentration reliably induced hallmark features of DED, including significant corneal fluorescein sodium staining, basal epithelial vacuolisation, and a marked reduction in conjunctival goblet cells (40, 41). These pathological and histological alterations collectively confirm the robustness of the model and provide a reliable platform for evaluating therapeutic interventions targeting DED pathophysiology.

The gut microbiota possesses extensive metabolic capabilities and can expand the host’s utilisation of nutrients, playing a critical role in maintaining health and resisting disease (42). Retinoic acid, the principal active metabolite of VA, has been demonstrated in multiple studies to effectively alleviate DED (9, 43). Previous research has shown that gut microbes with aldehyde dehydrogenase activity, such as *L. intestinalis*, can metabolise VA to RA and modulate the gut microbial community, thereby enhancing the host’s ability to produce RA from VA (24, 37). A study on *B. cereus* ALDH sequences demonstrated this RA-producing capacity, and similar approaches have been extended to bifidobacteria (25, 27). Using this strategy, we identified *B. breve* CCFM1489 as possessing a highly homologous ALDH sequence and capable of producing RA from VA *in vitro*. As research on the gut–eye axis progresses, it has become evident that gut microbes can influence various ocular diseases (44, 45). Therefore, probiotic interventions using RA-producing gut bacteria to modulate the gut microbiota may represent an effective approach to mitigate DED. This conceptual framework directly motivated the experimental design of the present study.

Using the BAC-induced DED model, we demonstrated that *B. breve* CCFM1489 effectively alleviated DED, comparable to direct RA supplementation and superior to VA administration alone. CCFM1489 treatment significantly improved corneal epithelial integrity, reduced ocular surface damage, and restored conjunctival goblet cell density. These improvements were accompanied by reduced inflammatory and oxidative stress markers, supporting a systemic rather than purely local mechanism of action.

Importantly, CCFM1489 administration significantly elevated RA levels in bothfeces and serum, indicating enhanced intestinal conversion and systemic availability of retinoids. Given that RA is the principal bioactive metabolite responsible for VA-mediated epithelial homeostasis and immune regulation, these findings suggest that the therapeutic efficacy of CCFM1489 is closely linked to its capacity to augment endogenous RA production rather than simple VA delivery (46). VA supplementation alone failed to produce robust therapeutic effects despite increased substrate availability. This finding underscores the importance of microbial bioconversion in determining VA bioactivity. Without sufficient ALDH-expressing gut microbes, dietary VA may remain poorly converted to RA, limiting its systemic and ocular efficacy. These results highlight the gut microbiota as a critical determinant of VA bioavailability and suggest that probiotic-assisted retinoid metabolism may be more effective than vitamin supplementation alone. These findings suggest that the gut microbiota may play an important role in determining VA bioavailability, and that probiotic-assisted retinoid metabolism may offer advantages over vitamin supplementation alone. However, direct causal relationships remain to be established.

Mechanistic insights were further explored through metabolomics and metagenomics. From a microbiome perspective, CCFM1489 intervention reshaped gut microbial composition and enriched specific microbial taxa. Notably, *L. plantarum* abundance increased significantly following CCFM1489 treatment. *In vitro* assays confirmed that *L. plantarum* is capable of metabolising VA into RA, suggesting it may contribute to the elevated RA levels observed in the gut (Supplementary Figure S6).

Additionally, both CCFM1489 and RA interventions enriched *P. goldsteinii*, a species known for its anti-inflammatory properties and capacity to produce short-chain fatty acids (SCFAs), particularly propionate and butyrate (47, 48). These SCFAs are known to enhance epithelial barrier function, modulate immune responses, and improve lipid absorption, collectively creating a metabolic environment conducive to efficient retinoid uptake and systemic distribution (47, 48). While the direct functional roles of these taxa in RA metabolism remain to be fully established, their enrichment alongside other metabolic improvements suggests they may represent potentially beneficial taxa associated with the observed effects. Further studies using gnotobiotic models are required to definitively elucidate their contributions.

Consistent with microbiome alterations, faecal metabolomics revealed that CCFM1489 intervention significantly enriched linoleic acid metabolism pathways, which are closely associated with the absorption of fat-soluble vitamins, including retinoids (49). Conjugated linoleic acid (CLA) isomers, particularly c9,t11-CLA, have been shown to enhance intestinal absorption and bioavailability of retinoid metabolites, providing a plausible mechanistic explanation for the elevated systemic RA levels observed in the CCFM1489 group (49). Moreover, enrichment of unsaturated fatty acid biosynthesis and tryptophan metabolism the CCFM1489 intervention. Elevated levels of docosahexaenoic acid (DHA) and eicosapentaenoic acid (EPA), both of which possess anti-inflammatory and reactive oxygen species–scavenging properties, may directly contribute to ocular surface protection and symptomatic improvement in DED (Supplementary Table S4) (50). These lipid mediators have been previously implicated in the stabilization of the tear film and the suppression of ocular inflammatory responses, raising the possibility that CCFM1489 confers protective effects through pleiotropic metabolic reprogramming that extends beyond retinoic acid-mediated pathways. In line with this hypothesis, treatment with CCFM1489 was associated with a significant elevation of intestinal tryptamine levels, suggesting potential crosstalk between tryptophan-derived metabolites and retinoid metabolism. While direct mechanistic evidence linking tryptamine to VA metabolism remains limited, existing studies suggest a positive association between tryptamine and retinol levels, warranting further investigation into potential crosstalk between tryptophan-derived metabolites and retinoid pathways (51).

Furthermore, Among shared differential metabolites across interventions, indolelactic acid emerged as a key molecule restored to control levels following CCFM1489, VA, or RA treatment. Correlation analysis revealed positive associations between indolelactic acid and enriched *L. plantarum* and *P. goldsteinii*, consistent with the known capacity of *L. plantarum* to convert tryptophan into indolelactic acid (52). Indolelactic acid is a recognised microbial ligand of the aryl hydrocarbon receptor (AHR), a pathway implicated in epithelial barrier maintenance and immune modulation. Previous studies suggest that AHR activation, particularly in the presence of *Akkermansia muciniphila*, may enhance host RA synthesis (53). Although *A. muciniphila* abundance remained relatively stable across all groups in the present study, the concomitant elevation of indolelactic acid suggests a potential permissive role for this metabolite in facilitating host retinoid metabolism. However, this proposed mechanism remains speculative and requires direct experimental validation, such as AHR inhibition or receptor-specific knockout models.

Despite leveraging metagenomics and untargeted serum and faecal metabolomics to explore differential pathways and potential mechanisms, the interactions amongst RA-producing gut microbes and their regulatory effects on the host remain to be fully elucidated.

Previous probiotic-based interventions for dry eye disease have primarily focused on anti-inflammatory and immunomodulatory mechanisms. Yun et al. demonstrated that *Lactobacillus plantarum* and *B. bifidum* alleviated dry eye in mice by modulating gut inflammation and microbiota (4). Similarly, a combination of lactoferrin, fish oil, and *E. faecium* WB2000 was shown to relieve dry eye symptoms in both rodent models and clinical populations (21). The probiotic consortium IRT5 (*B. bifidum*, *L. acidophilus*, *L. casei*, *L. reuteri*, and *S. thermophilus*) ameliorated autoimmune dry eye disease in mice (22). While these studies established the potential of probiotics to influence ocular surface health through the gut-eye axis, none of them specifically targeted vitamin A metabolism as a mechanistic strategy. In contrast, our study is distinguished by screening for ALDH-active strains with specific vitamin A-to-retinoic acid conversion capacity, thereby directly enhancing endogenous RA production rather than merely modulating inflammatory responses. This mechanism-based approach offers a paradigm shift from conventional immunomodulatory probiotic interventions to metabolism-targeted strategies for DED management.

Several limitations of this study should be acknowledged. First, our current experimental design does not allow us to definitively determine whether the observed microbial alterations are directly caused by CCFM1489 supplementation or indirectly result from the improvement of disease status, as these two possibilities cannot be distinguished in the present correlational study. The enrichment of specific taxa may reflect either direct modulation by CCFM1489 or indirect shifts associated with the attenuation of inflammation and oxidative stress in the host. Second, the intervention period was relatively short (7 days), which limits our ability to assess the long-term efficacy and safety of CCFM1489 supplementation. Third, the BAC-induced dry eye mouse model, while widely used, does not fully replicate the complexity of human DED, and caution should be exercised when extrapolating our findings to clinical settings. Fourth, our microbiome and metabolomics analyses are correlative in nature and do not demonstrate causality. Future studies using germ-free or gnotobiotic mouse models, combined with fecal microbiota transplantation, would be valuable to establish causal relationships between specific microbial taxa and the observed therapeutic effects.

Although our results demonstrate that direct oral RA supplementation is effective in alleviating DED symptoms, it is also important to recognize the potential systemic adverse effects of this approach. RA is a potent teratogen and can cause significant side effects—such as mucocutaneous dryness, headache, and hepatotoxicity—when administered at high doses or over prolonged periods systemically. However, it should be noted that the RA intervention in this study involved short-term administration (7 days, 0.5 mg/day), a regimen well below the doses and durations associated with significant toxicity in rodent models reported in the literature. Such brief exposure is unlikely to induce the severe adverse reactions observed with chronic or high-dose RA treatment. Probiotics like CCFM1489, which enhance endogenous RA production, may achieve therapeutic effects through the host’s own regulatory mechanisms, offering a safer alternative that avoids the risks associated with supraphysiological doses or long-term RA supplementation. This highlights a key clinical advantage of our microbiota-targeted strategy.

## 5 Conclusion

Oral administration of *B. breve* CCFM1489, a probiotic strain with high VA-to-RA metabolic capacity, alleviates BAC-induced DED by reducing corneal epithelial damage and goblet cell loss whilst elevating RA levels in DED mice. CCFM1489 modulates corneal antioxidant enzyme activities and pro-inflammatory cytokine levels, concurrently enriching specific gut microbes including *L. plantarum* and *P. goldsteinii*, which possess potential RA-producing capabilities, thereby restoring gut microbiota structure. This study provides novel insights into the beneficial effects of probiotics in alleviating DED and will facilitate future identification and evaluation of RA-producing probiotic strains for ocular health applications.

## Data Availability

The datasets presented in this study can be found in online repositories. The names of the repository/repositories and accession number(s) can be found below. The raw metagenomic sequencing data have been deposited in the NCBI SRA database under BioProject accession number PRJNA1518375. The metabolomics data have been deposited in the MetaboLights repository under accession numbers MTBLS15449 and MTBLS15440.
