# Supplementary material for: Bifidobacterium breve alleviates dry eye disease by regulating gut microbiota and vitamin A metabolism
Source: Front Nutr. 2026 Sep 9;13:1956357. doi: 10.3389/fnut.2026.1956357 (PMC13597888; doi:10.3389/fnut.2026.1956357)

Supplementary Materials

S1 BLASTP comparison of ALDH across different Bifidobacterium breve strains.

Strain	Identity (KFL74159.1)	E.value (KFL74159.1)	Identity (WP_015438559.1)	E.value (WP_015438559.1)
CCFM1489	26.303 %	8.29e-21	98.978 %	0
FAHWH21M7	26.464 %	2.83e-21	99.422 %	0
FGZ39MM6	28.956 %	8.70e-18	96.822 %	0
FFJND11M5	26.464 %	2.32e-09	98.844 %	0
FXJCJ32M7	26.464 %	2.47e-09	99.037 %	0
FNMHLBE8M6	26.464 %	2.47e-09	99.422 %	0
FHuNaL441	26.464 %	5.35e-21	99.037 %	0
FGZ23I1M6	26.355 %	8.73e-21	98.984 %	0
FWX346	27.353 %	1.41e-20	98.554 %	0

S2 OPLS-DA analysis showing feces metabolic differences between intervention groups and the MOD group.

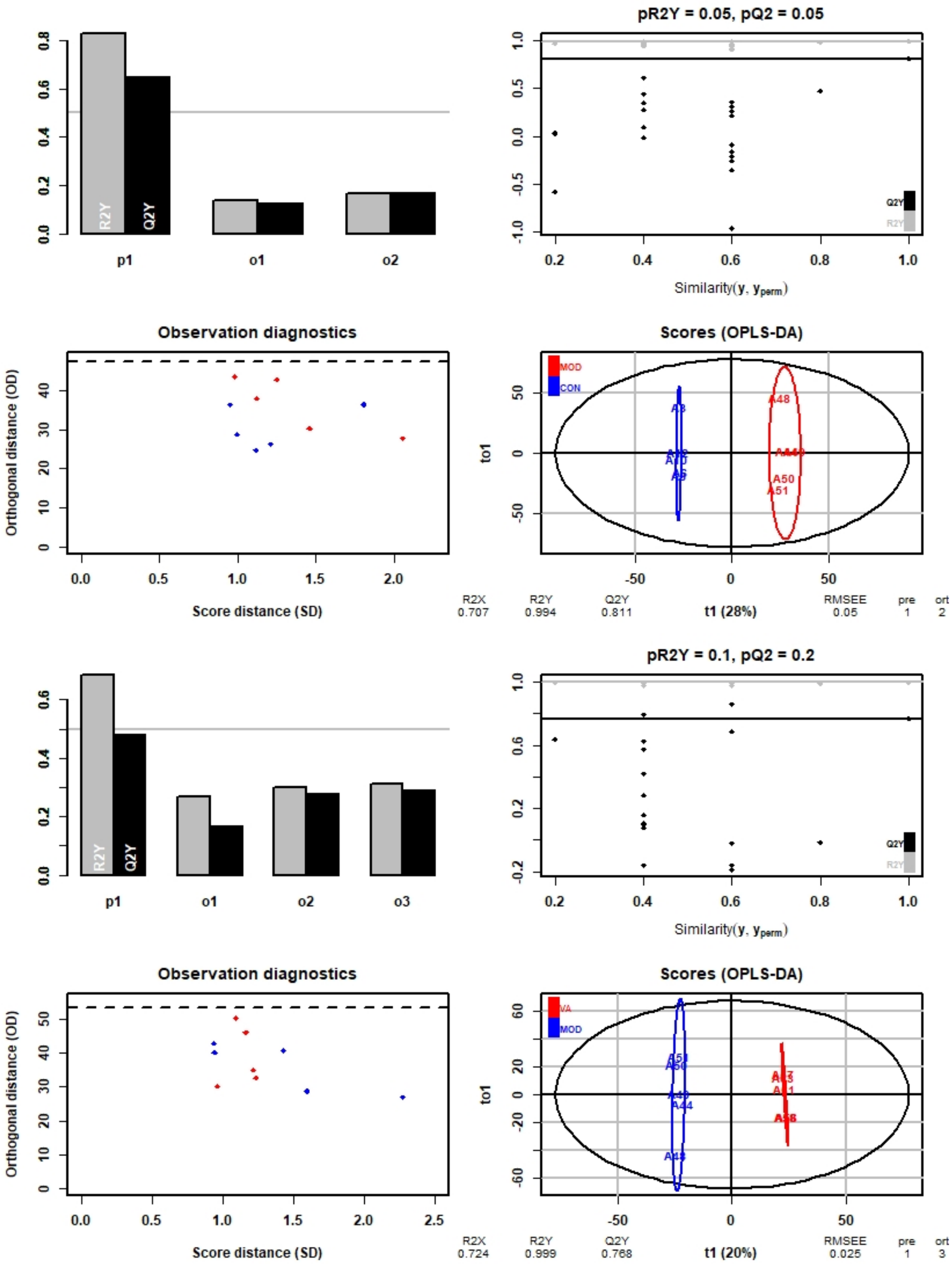

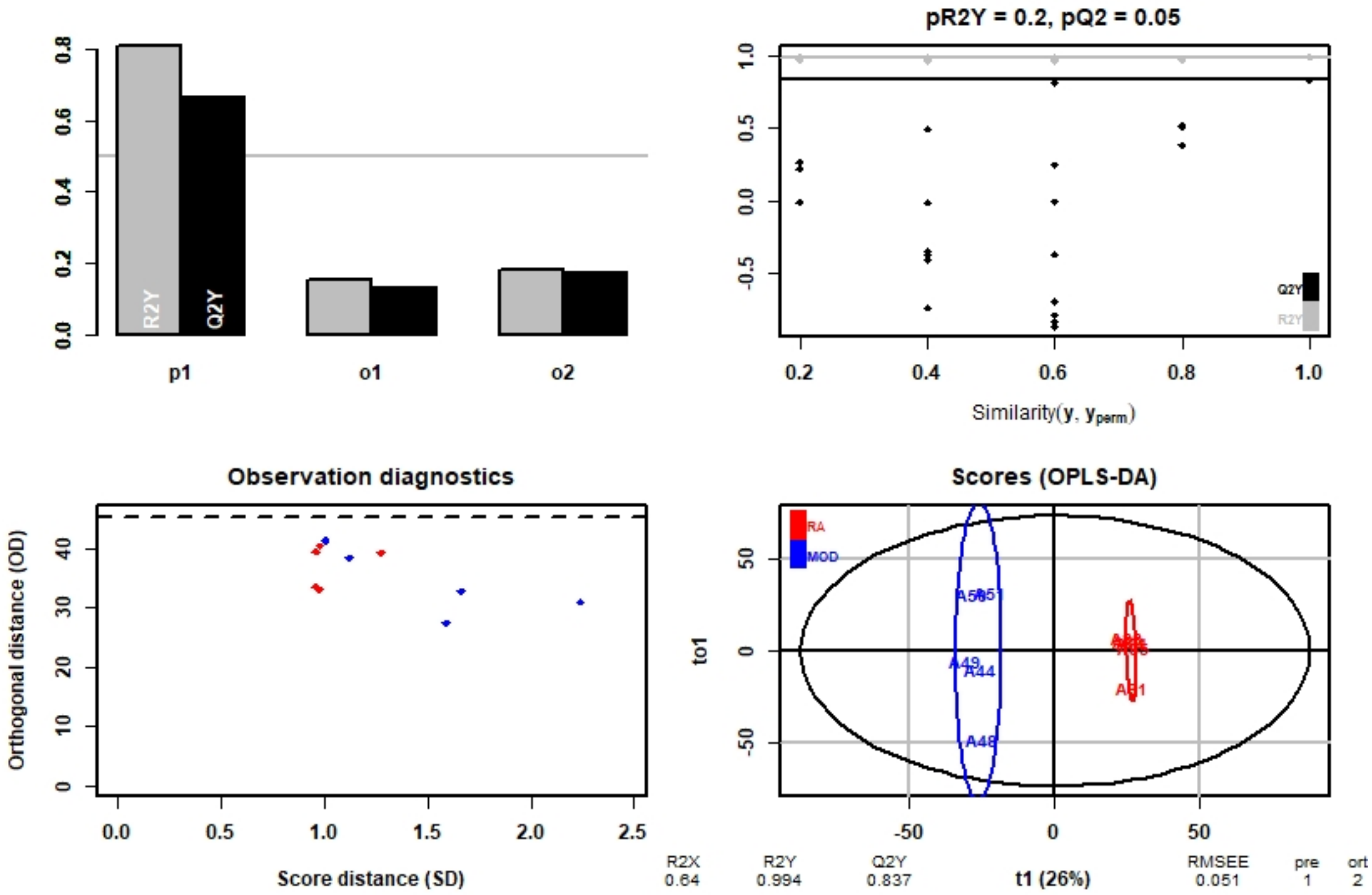
pR2Y = 0.2, pQ2 = 0.05
Similarity(y, yperm)
Observation diagnostics
Orthogonal distance (OD)
Score distance (SD)
Scores (OPLS-DA)
RA
MOD
to1
t1 (26%)
R2X 0.64
R2Y 0.994
Q2Y 0.837
RMSEE 0.051
pre 1
ort 2

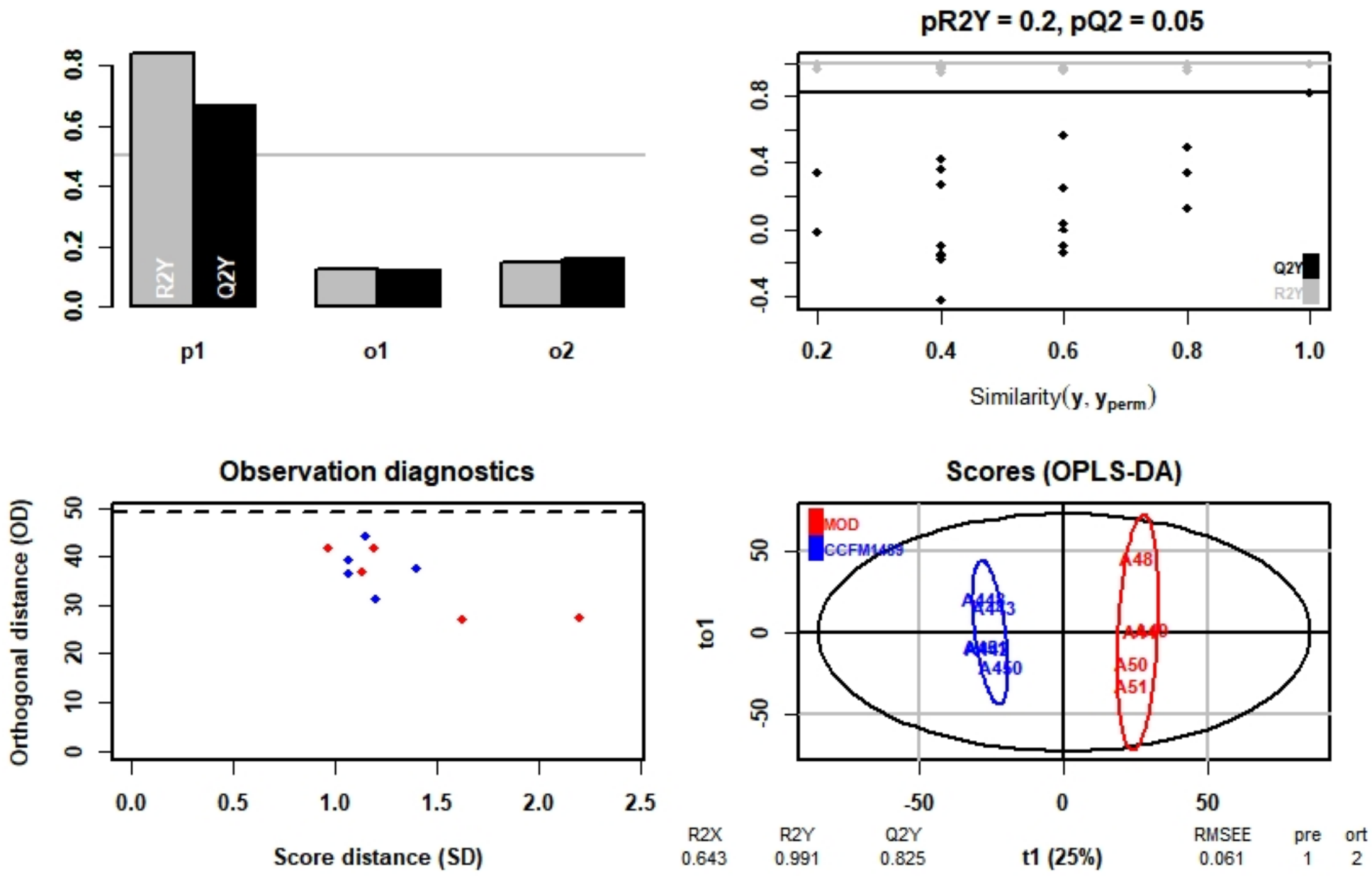
pR2Y = 0.2, pQ2 = 0.05
Similarity(y, yperm)
Observation diagnostics
Orthogonal distance (OD)
Score distance (SD)
Scores (OPLS-DA)
MOD
CCFM1489
to1
t1 (25%)
R2X 0.643
R2Y 0.991
Q2Y 0.825
RMSEE 0.061
pre 1
ort 2

S3 PLS-DA analysis showing serum metabolic differences between intervention groups and the MOD group.

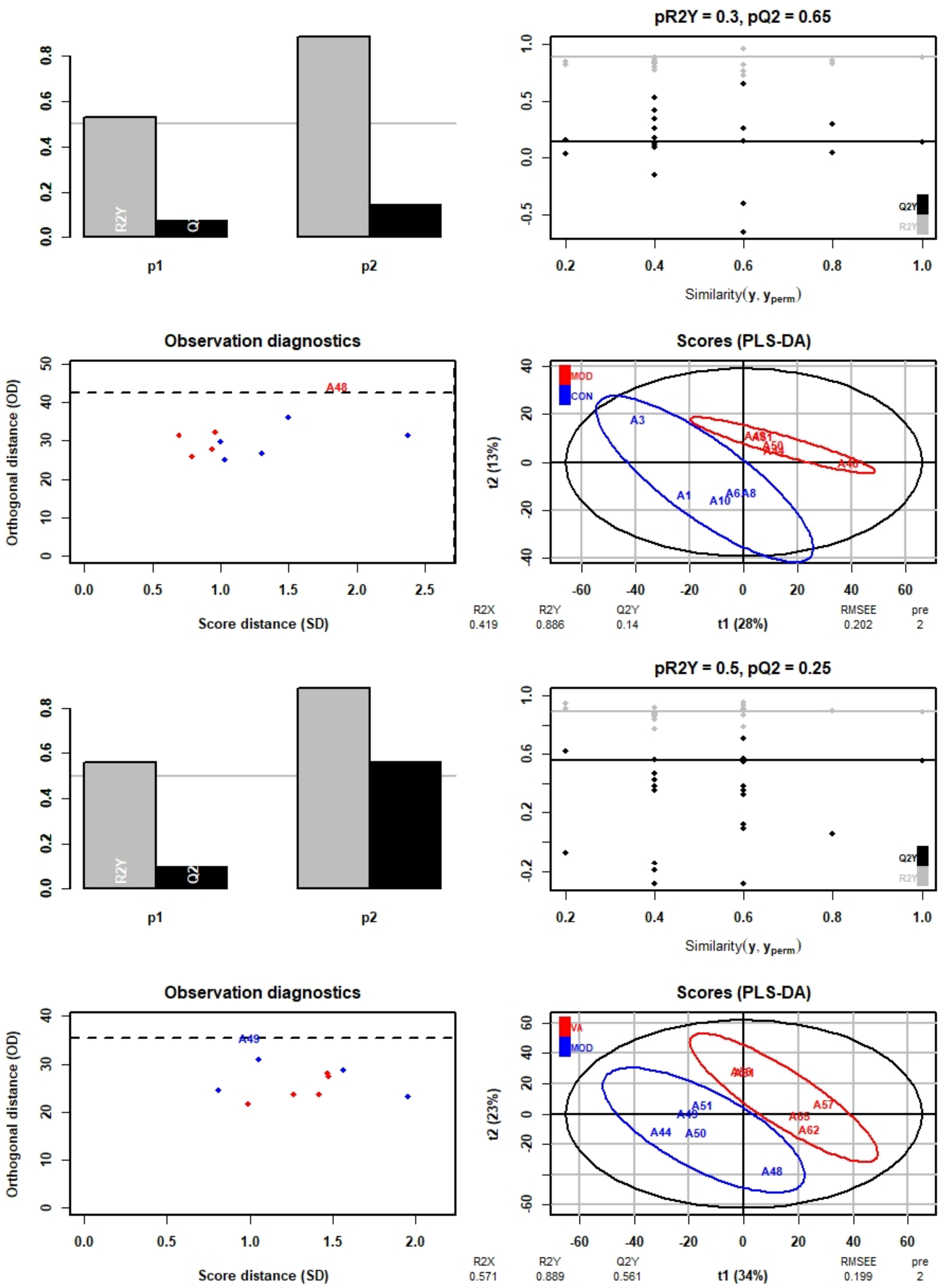

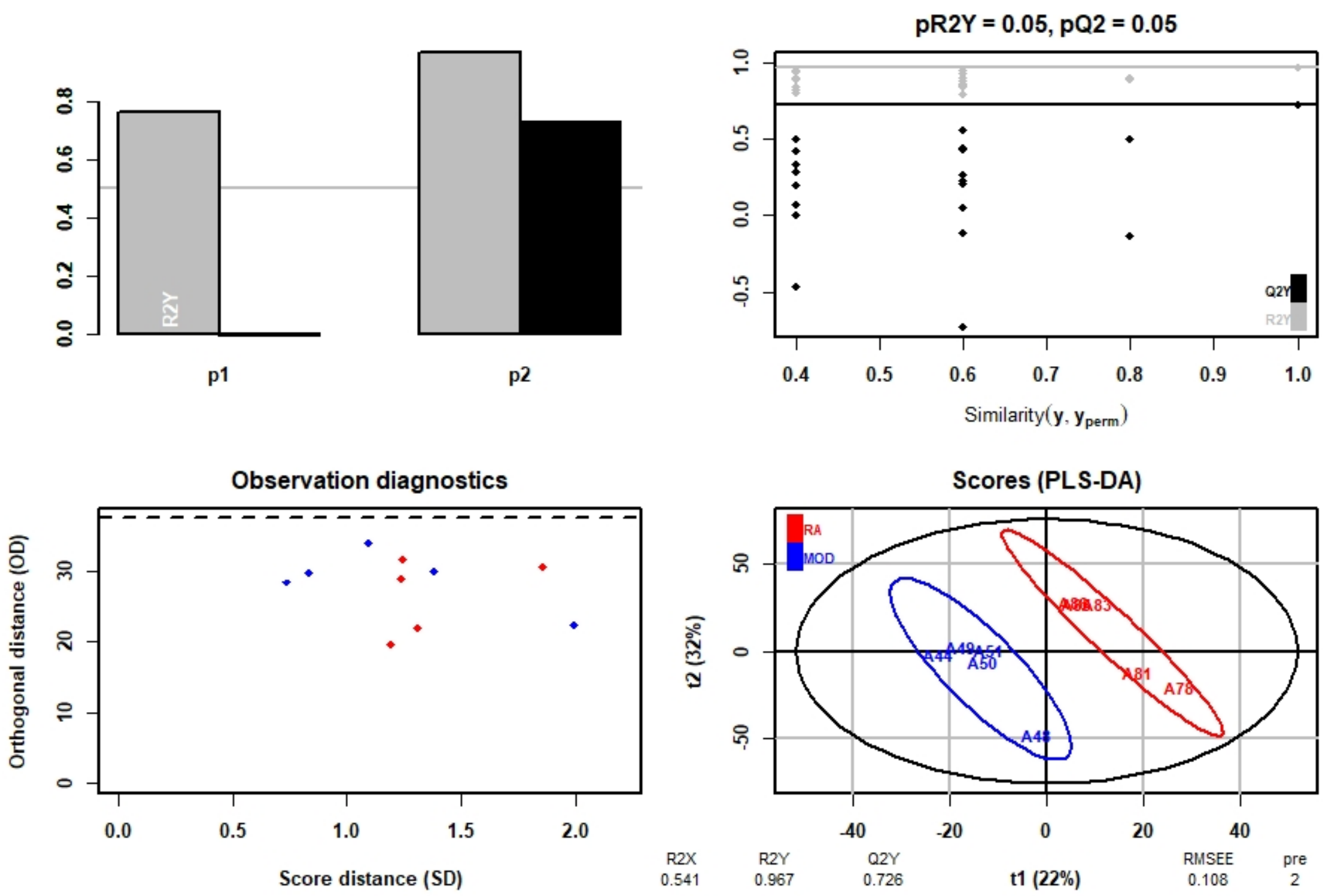

pR2Y = 0.05, pQ2 = 0.05
R2Y
p1
p2
Q2Y
R2Y
Similarity(y, yperm)
Observation diagnostics
Orthogonal distance (OD)
Score distance (SD)
Scores (PLS-DA)
RA
MOD
t2 (32%)
t1 (22%)
A44
A49
A51
A50
A48
A81
A78
A83
R2X 0.541
R2Y 0.967
Q2Y 0.726
RMSEE 0.108
pre 2

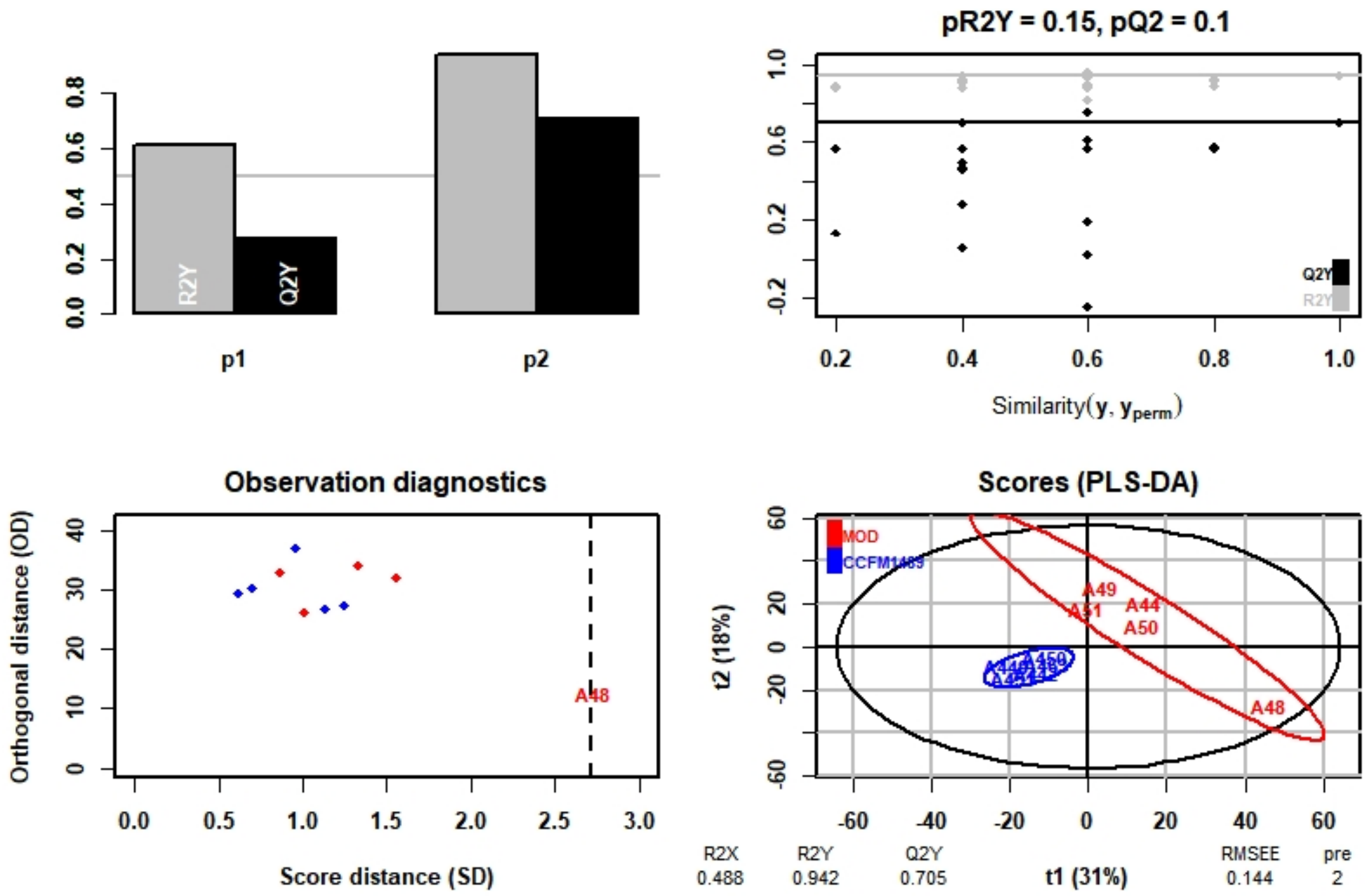

pR2Y = 0.15, pQ2 = 0.1
R2Y
Q2Y
p1
p2
Q2Y
R2Y
Similarity(y, yperm)
Observation diagnostics
Orthogonal distance (OD)
Score distance (SD)
A48
Scores (PLS-DA)
MOD
CCFM1489
t2 (18%)
t1 (31%)
A49
A51
A44
A50
A48
R2X 0.488
R2Y 0.942
Q2Y 0.705
RMSEE 0.144
pre 2

S4 TOP 25 differential enriched fecal metabolic pathways in CCFM1489 and model mice

Total	Hits	Expect	P value	Holm P	Metabolites
Galactose Metabolism	38	5	1.37	0.00954	Galactitol, uridine diphosphate glucose, maltotriose, raffinose, stachyose
Alpha Linolenic Acid and Linoleic Acid Metabolism	17	3	0.611	0.0205	Eicosapentaenoic acid, docosahexaenoic acid, stearidonic acid
Retinol Metabolism	35	3	1.26	0.127	all-trans-retinoic acid，9-cis-retinal, 4-oxo-retinoic acid
Methylhistidine Metabolism	4	1	0.144	0.136	Histidine
Pyrimidine Metabolism	57	4	2.05	0.143	Deoxyuridine, thymidine, 5-thymidylic acid, dUMP
Vitamin B6 Metabolism	19	2	0.683	0.147	4-Pyridoxic acid, pyridoxamine 5'-phosphate
Tryptophan Metabolism	59	4	2.12	0.157	Kynurenic acid, xanthurenic acid, 4-(2-Amino-3-hydroxyphenyl)-2,4-dioxob utanoic acid, formylanthranilic acid
Purine Metabolism	73	4	2.62	0.264	Deoxyinosine, deoxyguanosine, guanosine, uric acid
Pyruvaldehyde Degradation	10	1	0.359	0.308	Glutathione;
D-Arginine and D-Ornithine Metabolism	11	1	0.395	0.333	2-Oxoarginine
Aspartate Metabolism	35	2	1.26	0.361	L-Arginine, N-Acetyl-L-aspartic acid
Lactose Synthesis	19	1	0.683	0.504	Uridine diphosphate glucose

Nucleotide Sugars Metabolism	20	1	0.719	0.522	Uridine diphosphate glucose
Glutathione Metabolism	20	1	0.719	0.522	Glutathione
Carnitine Synthesis	22	1	0.79	0.557	N6,N6,N6-Trimethyl-L-lysine
Phenylalanine and Tyrosine Metabolism	27	1	0.97	0.633	Phenylalanine
Urea Cycle	28	1	1.01	0.646	L-Arginine
Ammonia Recycling	31	1	1.11	0.684	Histidine
Starch and Sucrose Metabolism	31	1	1.11	0.684	Uridine diphosphate glucose
Bile Acid Biosynthesis	65	2	2.34	0.693	Lithocholyltaurine, taurochenodesoxycholic acid
Beta-Alanine Metabolism	34	1	1.22	0.718	Histidine
Nicotinate and Nicotinamide Metabolism	35	1	1.26	0.728	N1-Methyl-4-pyridone-3-carboxamide
Sphingolipid Metabolism	40	1	1.44	0.775	Uridine diphosphate glucose
Methionine Metabolism	42	1	1.51	0.792	Methionine sulfoxide
Histidine Metabolism	42	1	1.51	0.792	Histidine

S5 TOP 8 differential serum metabolic pathways in CCFM1489 and model mice

Total	Hits	Expect	P value	Holm P	Metabolites
Phenylalanine, tyrosine and tryptophan biosynthesis	34	1	0.031	0.0307	Shikimic acid
Xylene degradation	38	1	0.0346	0.0342	4-Isopropylbenzoic acid
Drug metabolism - other enzymes	39	1	0.0355	0.0351	5-Fluorouridine
Biosynthesis of amino acids	125	1	0.114	0.11	Shikimic acid
Degradation of aromatic compounds	210	1	0.191	0.179	4-Isopropylbenzoic acid
Microbial metabolism in diverse environments	800	1	0.729	0.566	4-Isopropylbenzoic acid
Biosynthesis of secondary metabolites	913	1	0.832	0.623	Shikimic acid
Metabolic pathways	2150	2	1.96	0.721	Shikimic acid;4-Isopropylbenzoic acid

S6 *Lactiplantibacillus plantarum* converts vitamin A into retinoic acid

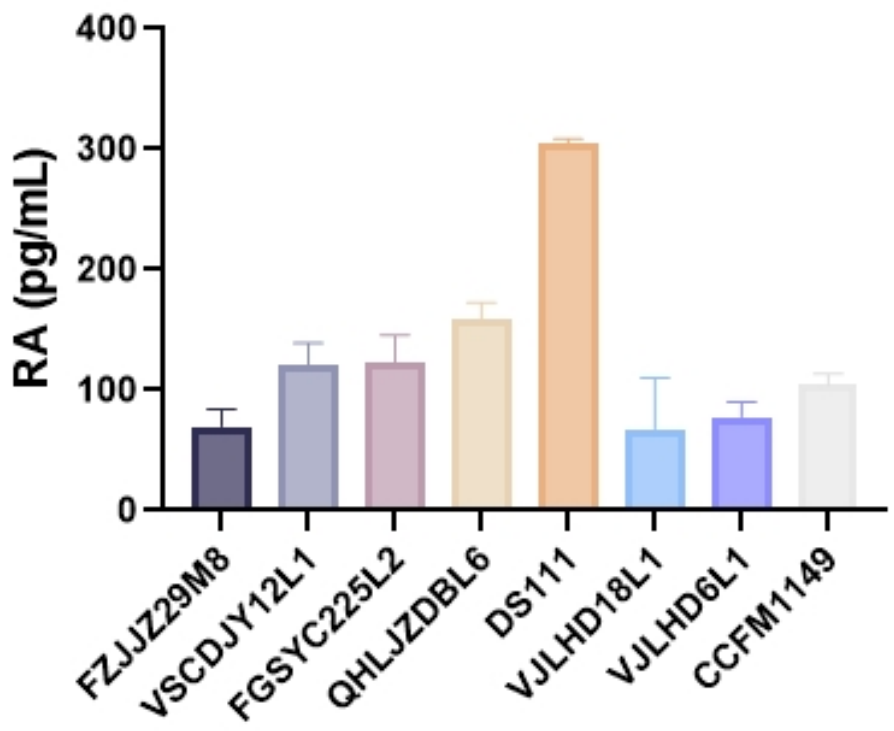

Supplement: Supplementary file 1 [file Data_Sheet_1.pdf]
